# Nanoscale imaging of E. coli cells by expansion microscopy

**DOI:** 10.15190/d.2019.11

**Published:** 2019-09-30

**Authors:** Sharey Cheng, Yongxin Zhao

**Affiliations:** Department of Biological Sciences, Carnegie Mellon University, Pittsburgh, PA, USA

**Keywords:** Expansion Microscopy, Escherichia coli, bacteria, super-resolution imaging.

## Abstract

Expansion microscopy (ExM) is an emerging super-resolution imaging technology. ExM works by infusing a biological specimen with a superabsorbent hydrogel, followed by mechanical homogenization and isotropical expansion of the specimen in water. The unique and cost-effective process of ExM enables super-resolution optical imaging of sample of interest without the need to invest and use of a sophisticated microscope instrument. Here, we demonstrate that a nearly 3-fold isotropic physical expansion of E.coli fixed cells can be achieved in PBS, and the cell morphology during binary fission is clearly resolved in the expanded state, using a diffraction-limited microscope.

Expansion microscopy (ExM) is a newly emerged imaging technique that seeks to overcome the everlasting hindrance of the optical imaging system, the diffraction limit. Different from the other super-resolution microscopy types (for example Structured illumination microscopy (SIM), Stochastic optical reconstruction Microscopy (STORM) or Stimulated emission depletion microscopy (STED)), ExM provides the solution to achieve nano-scale resolution (~70 nm) through the physical expansion of biological samples, independent of an intricate imaging system^1^. The fundamental of the physical expansion of biological specimens is to infuse fixed samples with the gelling solution consisting of monomers, followed by *in situ *polymerization for the formation of a tissue hydrogel hybrid. The gelling solution consists of sodium acrylate, a monomer that confers superabsorbent properties, along with co-monomer acrylamide, cross-linker N-N′-methylene-bisacrylamide, accelerator tetramethylethyl-enediamine, and initiator ammonium persulfate, to trigger the free-radical polymerization. After homogenizing the mechanical properties of the sample-gel composite, isotropic expansion can be achieved after iterative washes with pure water. The extent of expansion, which is defined as expansion factor, can be controlled by varying the salt concentration of the solution, where the maximum expansion factor is reached with pure water.

Parameters in the steps of gelation and homogenization and the strategies for presenting target molecules have been continually improving to accommodate a broader range of applications. The first version of ExM requires the unique design of the fluorescent labels that can directly incorporate to the polymer network to survive the homogenization with protease K^2^. The protein-retention ExM (proExM) later managed to preserve antigens in the gel via modification of the amines on proteins with an acrylamide functional group that participate in the free-radical polymerization for anchoring^3^. Successful expansions have been performed among a wide range of cell lineages, and tissue types^4^, showing the potential of ExM to facilitate the basic science research and clinical sample diagnosis.

Owing to the small size, microbial cells, such as bacteria and fungi, are challenging to study using conventional optical microscope. Nanoscale optical imaging of microbial cells can potentially open new doors to experimental observation of novel biological structure with molecular contrast, which is not available with electron microscopy. Escherichia coli (*E. coli*) is one of the most studied gram-negative bacteria. *E. coli* possesses a cell wall composed of a thin peptidoglycan layer (2-4 nm) and an outer membrane layer. The peptidoglycan polysaccharide chain is composed alternatively from N-acetylglucosamine and N-acetylmuramic acids and is further crosslinked by tetrapeptides, exhibiting rigidity for maintaining cell shape and providing protection from osmotic lysis^5^. In a recent study, application of ExM on imaging *E. coli* cells has been reported in combination with a miniaturized microscope^6^, using the first generation ExM^2^.

Here, we demonstrate the application of ExM on imaging *E. coli* cells with ~80 nm resolution. *E. coli *TOP10 cells, a common chemical competent *E. coli *strain, were fixed with 4% paraformaldehyde (PFA) and stained with DAPI (4′,6-diamidino-2-phenylindole) and a lectin wheat germ agglutinin (WGA) to visualize its nucleus and polysaccharide-containing cell structure, respectively (**Figure 1**). Images were allacquired with a Nikon Ti2 eclipse fluorescence microscope, with a CSU-W1 confocal module under a long working distance water immersion 40× objective (numerical aperture (NA) = 1.33, confocal microscope). Without expansion it is hard to clearly distinguish single cells and both DAPI and WGA stains overlapped within a rod shape (**Figure 1A, C**). The treatment of enzymatic homogenization allows isotropic expansion of the gelled *E. coli* cells. Approximately 3-fold expansion factor (2.8) was achieved in PBS (**Figure 1B**). After expansion, the cell morphology during binary fission is clearly resolved while the overall distortion compared to pre-expansion state is minimal (**Figure 1D**). Further expansion is achievable with pure water, if additional resolving power is needed.

**Figure 1:**
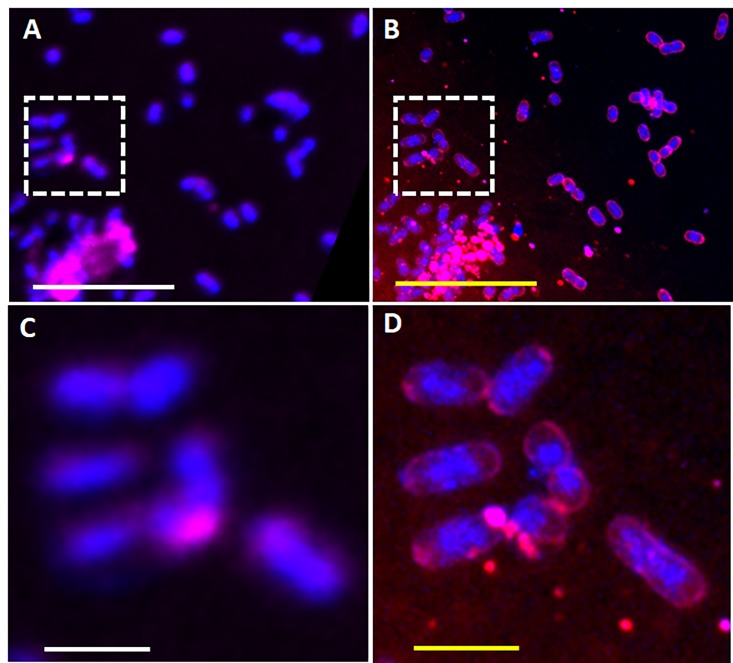
Nanoscale imaging of E. coli cells by expansion microscopy. **A.** Image of pre-expansion E.coli TOP10 cells. Stain: DAPI, Blue; WGA, Magenta. **B.** Image of expanded E.coli cells in the same field of view as A.** C.** Zoom-in field of view of A as indicated by white dash box. **D.** Zoom-in field of view of B as indicated by white dash box. Scale bar (in biological scale): (**A-B.**) 10 μm; (**C-D.**) 2 μm. Expansion factor: 2.8 in 1× PBS.
